# Research on the association between community environmental perception and residents’ intention to relocation–a case study of Guangzhou

**DOI:** 10.3389/fpubh.2025.1557220

**Published:** 2025-06-20

**Authors:** Jie Gu, Chunxia Zhang, Xiaoxue Li, Xiao Chen

**Affiliations:** ^1^College of Architecture and Urban Planning, Key Laboratory of Key Technologies of Digital Urban-Rural Spatial Planning of Hunan Province, Hunan City University, Yiyang, China; ^2^Center of Online Behavior Security Education and Research for College Students, School of Tourism and Business, Guangzhou Polytechnic University, Guangzhou, China; ^3^Center of GeoInformatics for Public Security, School of Geography and Remote Sensing, Guangzhou University, Guangzhou, China

**Keywords:** residential relocation intention, community environmental perception, factor analysis, disorderly environment, Guangzhou

## Abstract

**Background:**

The community environment is an important factor affecting people’s residential relocation; however, existing literature has primarily focused on the objective aspects of the community environment, with less emphasis on residents’ perception of it.

**Method:**

To address this research gap, we selected 74 typical communities and collected 1,568 questionnaires across Guangzhou. We employed factor analysis to capture participants’ community environmental perception and used binary logistic regression to analyze the association between independent and dependent variables.

**Results:**

The results show that: (1) There is a significant association between age, household registration, and participants’ residential relocation intention; (2) Community environmental perception can be summarized into three aspects: environmental disorder perception, community attachment, and satisfaction, all of which are significantly associated with participants’ residential relocation intention; and (3) There is a positive association between perception of a disorderly environment and residents’ intention to relocation, and a negative association between community attachment and satisfaction and residents’ intention to relocation.

**Conclusion:**

This research is highly significant for enhancing our understanding of factors influencing people’s residential relocation intention and for guiding community construction.

## 1 Introduction

### 1.1 The definition of residential relocation

The literature on residential relocation acknowledges that residential relocation should be distinguished from migration (1). Residential relocation can be defined as a displacement of the place of residence, triggering complementary action with respect to transportation and not to the relocation of other forms of activities (2). Migration can be defined as a multiple relocation decision with respect to more than just the place of residence (3). In this paper, the research on residential relocation refers to short-distance moves not exceeding the boundaries of the daily activity space. Therefore, this paper uses the concept of residential relocation rather than migration. Residential relocation results from the interaction of many complex factors (4). The community environment of the current residence undoubtedly has an important influence on residential relocation. However, do residents’ community environmental perception affect residential relocation? This question is not only interesting but also serves as a positive inspiration for the community environment.

### 1.2 Influencing factors of residential relocation

The academic research on residential relocation can generally be summarized into three types (5). The first type of study attributes the causes of residential relocation to individual characteristics. In terms of age, a nonlinear association between age and residential relocation has been reported (6, 7). For employment status, a significant association between employment status and residential relocation has also been reported; comparatively speaking, an unemployed individual is more likely to move than an otherwise similar employee (8). In terms of socioeconomic attributes, a study found that the health of men and women moving from high to low mortality districts could be explained by the advantage over their lifetimes (9). In terms of household income, women from the lowest income level were more likely to move than those from the highest income level during pregnancy (10).

The second type of factor that explains residential relocation is life events. Moving from one place to another is an important life event accompanied by personal short-and long-term changes in life, and thus can be explained from the perspective of important life events (11). Some studies have focused on the relationship between family formation and home ownership and identified a association between first birth and residential relocation (12, 13). In addition to the birth of a child, other life node events that affect residential relocation include marriage, divorce, the death of a family member, and the acquisition of important job opportunities (14).

The third type of factor is cultural and social preferences. De Jong argues that relocation decision-making should consider three classes of variables (individual human capital attributes, household characteristics, and community characteristics) and that expectations along with family norms about relocation are major predictors of intention to move, which in turn is a proximate determinant of relocation behaviors (15). Other studies suggest that satisfaction with the destination, origin household, and neighborhood is another factor affecting residential relocation (16–18).

Residential relocation has been a frequently explored topic in geography and other disciplines. In terms of geography, resident relocation is the micro-driving force for the reconstruction of urban spatial structure (19). For demography, residential relocation is an important factor in explaining the law of population development (20). For urban planning, the change in the supply and demand relationship between infrastructure and public service facilities caused by residential relocation is a key factor in urban planning and design (21). As for traffic, residential relocation influences traffic organization through the intermediary factor of the job-residence relationship (22). For urban management, residential relocation has an important impact on the housing demand in different areas of the city and affects many aspects of urban housing management, such as housing price and property rights structure (23).

### 1.3 Community environmental perception as an factor of residential relocation

China has gone through very rapid urbanization and market-oriented reform of the housing system. From the founding of the People’s Republic of China in 1949 to the 1980s, China implemented the housing distribution model in urban areas under the planned economy system. After about 20 years of exploration and practice, China issued the Notice of The State Council on Further Deepening the Reform of the Urban Housing System and Accelerating Housing Construction in 1998 (24). This policy proposed stopping the physical distribution of housing and gradually realizing the commercialization reform of housing distribution, marking China’s departure from welfare housing distribution at the institutional level.

However, the rapid development of the real estate market has also brought about high housing prices and speculation. To this end, China planned and issued requirements for “accelerating the establishment of a housing system featuring multi-entity supply, multi-channel support, and a combination of rental and purchase.” The state made it clear that it would adhere to the basic position that “houses are for living in and deployed for speculation” so that all the people would have adequate housing. The state has clarified the basic positioning of housing: “houses are for living in, not for speculation,” aiming at providing housing for all people.

The residential relocation of urban residents in China is subject to national policies, residents’ economic conditions and rigid demands. Therefore, scholars focus more on these factors. The individual’s community environmental perception and emotional preference are generally ignored in the decision-making process of residents’ residential relocation in the real process and relevant research. With the further development of China’s economy and the superposition of various policies, such as the continuous development of the urban economy and real estate market, the deepening of housing monetization reform, and the introduction of various housing security policies, urban residents have been focusing more attention to the residential environment and individual emotional preferences in the process of residential relocation. Generally speaking, urban residents have more disposable income, which boosts real estate and increases security channels, providing them with more options on where to live. However, only a few studies have considered the impact of emotional factors, such as community environmental perception, on residential relocation.

Moreover, the psychological aspects of housing relocation are influenced by policy migration. Particularly, when the government implements related housing or land use policies, these policies often alter people’s relocation decisions and housing choices. A study on the southern neighborhoods of Memphis, Tennessee, suggests that the Choice Neighborhoods Initiative (CNI) had a positive impact on residents’ psychological attitudes toward housing relocation, as the CNI policy enhanced residents’ perceptions of family and community safety (25). Another study focusing on housing relocation for low-income women indicates that social policies can effectively address the psychological concerns of low-income women during relocation and improve their social withdrawal (26). Additionally, research on post-disaster forced housing relocation suggests that government interventions and related policies can significantly improve the psychological well-being of evacuees and reduce post-disaster housing instability (27). Not only urban areas, but rural residents’ relocation psychology is also influenced by policies. A study applying the “extended plan theory” examined how relocation poverty alleviation policies affected farmers’ willingness and behavior to relocate. The study found that farmers’ poor perception of the policy’s support after relocation hindered their willingness and actions to exit (28).

Community is the basic space unit of urban life and the main place for residents’ daily activities. Community environment can be divided into objective and subjective environments. Traditionally, research has distinguished between the objective and subjective aspects of the community environment. The objective environment refers to the built and living environments of the object. For example, Changes in the built environment affect residents’ daily travel (29) and changes in living environment can affect cardio-respiratory health (30) and lead to residential relocation, which have been widely documented to influence residential relocation decisions. In contrast, the subjective environment is an individual’s perception and emotion toward the objective environment. In the macro aspect, some scholars have realized the impact of the subjective environment of the community on the urban form and spatial structure (31). In the micro aspect, residential relocation not only affects and reshapes the urban spatial structure and form but also profoundly affects the subjective well-being of residents. In addition, environmental characteristics have significant effects on residents’ health behaviors (32). Therefore, the studies on the impact of subjective community environment on residential relocation intention are not only helpful in understanding the dynamics of urban spatial structure and form evolution from the micro level but also in providing a reference for environmental construction at the level of community planning. While some studies have acknowledged the role of subjective well-being and perceived neighborhood quality, they often treat these factors as supplementary or fragmented variables rather than integrating them into a coherent theoretical framework.

### 1.4 The present study

In this context, our study proposes a new analytical framework that explicitly structures subjective community environmental perception into three core dimensions: perceptions of environmental disorder, emotional attachment to the community, and overall community satisfaction. By systematically examining these interconnected components, we move beyond prior research that either narrowly focused on objective conditions or treated subjective experiences in isolation. Moreover, considering the unique socio-institutional dynamics in China—such as the Hukou system, rapid urban transformation, and evolving housing policies—subjective perceptions have become increasingly critical in influencing residents’ relocation intentions. This framework not only addresses gaps in the existing literature but also provides a nuanced lens to understand how emotional and cognitive evaluations of the community environment jointly shape residential mobility behaviors. Based on the literature review and research background, this study proposes the following testable hypotheses:

H1: Residents' higher perception of a disorderly community environment is positively associated with their intention to relocate.

H2: Stronger community attachment is negatively associated with residents' intention to relocate.

H3: Higher satisfaction with the community environment is negatively associated with residents' intention to relocate.

## 2 Data and methods

### 2.1 Data

The urban area studied in this paper is Guangzhou. Guangzhou is the third largest city in China and is located in the southern part of the country. The city comprises 11 districts: Tianhe, Yuexiu, Liwan, Haizhu, Baiyun, Panyu, Huangpu, Nansha, Huadu, Conghua, and Zengcheng. It has 2,806 neighborhood offices. According to the Statistical Yearbook of Guangzhou in 2022,^1^ the city’s administrative area covers 7434.40 square kilometers, Gross Domestic Product generated for the fiscal year came to USD 437.60 billion. The permanent population at year-end is 18.81 million; however, the number of registered permanent residents is only 10.12 million. Since China’s reform and opening up in 1978, the per capita housing construction in Guangzhou has rapidly grown. In 1980, the per capita housing construction area was about 4.00 square meters, while in 2021, it was 34.28 square meters.

**Figure 1:**
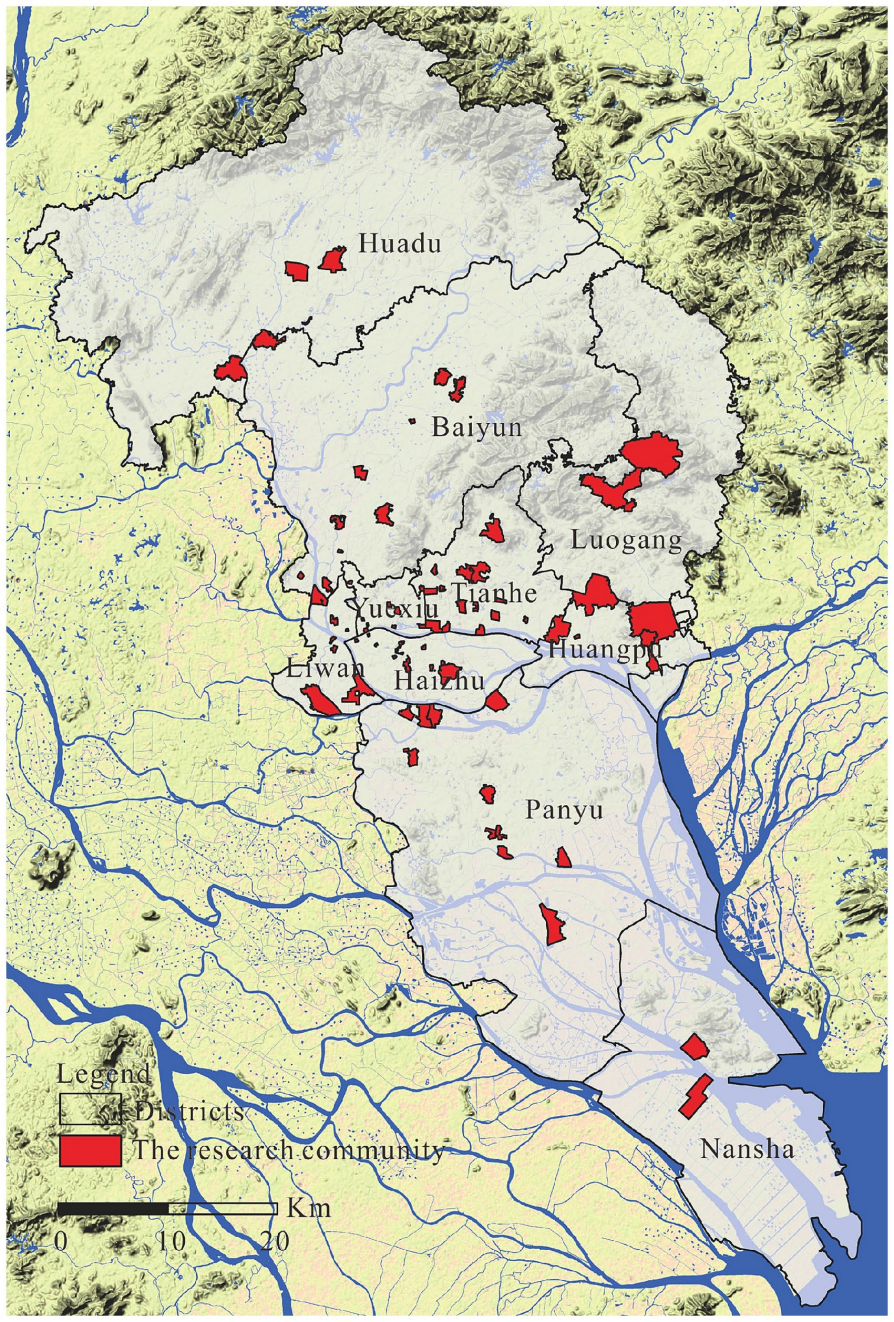
Communities that participated in the questionnaire.

Our data were obtained through a Questionnaire Survey of the Guangzhou Community Environment and Residents’ Safety Perception Project. The questionnaire survey was conducted from January to April 2016 (excluding the Spring Festival holiday), and its geographical scope included the entirety of Guangzhou city except for Conghua and Zengcheng Districts, which have relatively independent central urban areas and are located relatively far from the central urban area of Guangzhou. Firstly, With the six census data, we used the cluster analysis method to divide the communities in Guangzhou into nine social areas: middle-class, college, non-aging rural, old city aging, aging rural, migrant, low-rent housing, affordable housing, and high-end residential communities. Second, based on community classification and comprehensive consideration of the spatial distribution, 74 communities representing various social areas were selected using the stratified sampling method for the questionnaire survey after field investigation (Figure 1). Third, the questionnaire was distributed randomly, and the required interval of interviewees was more than five households. After the first round of investigation, the effectiveness of the questionnaire was tested, and a supplementary survey was conducted after eliminating unqualified questionnaires. Finally, 1,700 questionnaires were issued, and 1,568 valid questionnaires were collected, with an effective rate of 92.24%.

While the sample was drawn using stratified sampling across various types of communities to ensure diversity, it should be noted that strict proportional sampling based on the overall demographic structure of Guangzhou was not performed. To assess representativeness, we compared key sample characteristics with publicly available demographic statistics from the Guangzhou Statistical Yearbook (2022). Overall, the age distribution of the sample is slightly younger, the educational attainment level is moderately higher, and the household income is somewhat lower compared to the general urban population of Guangzhou. These differences should be considered when interpreting the results.

### 2.2 Measures

#### 2.2.1 Dependent variable

Dependent variables were obtained through a questionnaire survey. The question is “Do you plan to change your place of residence in the next year (within Guangzhou)?” with 1 for Yes and 0 for No was used.

#### 2.2.2 Independent variable

Independent variables include control and community environmental perception variables. Six control variables, gender, age, marital status, education, family income, and Hukou (household register), were used. Data were acquired directly from the questionnaire. Among the control variables, the first five are often used in residential relocation research, and a large collection of literature can be used for reference (33). The last variable is Hukou, which is very important in research on China-related topics (34, 35). Hukou is officially defined as a legal document recording the basic household population information, regulating population distribution and migration in China. China’s Hukou system is still very strict despite the loosening of some restrictions. A person who chooses to move to a city without a Hukou issued by the local government can legally live and work in the city but does not enjoy equal rights on public services, education, and healthcare compared to residents with local Hukou. Nowadays, the inequality of Hukou is largely reflected in the welfare of the local and non-local Hukou. Take education as an example; the education department divides school-age children into four levels: the first is those with a house and a household registration; the second is those with household registration but no house; the third is those with no household registration but a house; and the fourth those with no house and no household registration. The higher the grade, the higher the priority for the child to attend school. Therefore, a child who does not have a local Hukou will have considerable difficulty studying in a local school, especially in a public school. According to the Statistical Yearbook of Guangzhou in 2022, the non-local Hukou population in Guangzhou accounts for 46.23% of the permanent population. Therefore, Hukou is included as one of the control variables in this study.

This study measures residents’ community environmental perception from three dimensions: disorderly environment, community attachment, and satisfaction. Respondents were asked, “How do you agree with the following phenomena?.” Thirteen questions were designed to measure residents’ community environmental perception. Questions 1 to 4 are based on disorderly community environment, Questions 5 to 8 are based on emotional attachment to the community, and Questions 9 to 13 are based on community satisfaction.

The responses ranged from “I do not agree with that at all” to “I could not agree more” on a scale from 1 to 5. Table 1 shows that, on average, question 1 had the lowest score (average score: 2.761) and question 5 had the highest score (average score: 3.552).

**Table 1:** Measurement of community environmental perception and its rotated component matrix.

Model	Variable	Variable description	Mean	SD	*F*_1_	*F*_2_	*F*_3_
Model 1	Q1	Public facilities are often damaged.	2.761	0.962	0.744		
	Q2	Environment is disorderly (garbage, car parking)	2.961	0.992	0.857		
	Q3	Graffiti or posters are common in communities.	2.946	1.017	0.840		
	Q4	The neighborhood is very noisy.	2.930	0.984	0.806		
Model 2	Q5	I have a lot of affection for this community.	3.552	0.837		0.815	
	Q6	I have a lot of affection for my neighbors.	3.409	0.862		0.831	
	Q7	I love the architecture here.	3.386	0.825		0.833	
	Q8	I’m happy with the management of the community	3.452	0.787		0.826	
Model 3	Q9	I’m quite satisfied with the housing conditions.	3.430	0.772			0.769
	Q10	I’m quite satisfied with the enjoyable environment.	3.374	0.781			0.853
	Q11	I’m quite satisfied with the infrastructure.	3.346	0.771			0.844
	Q12	I’m quite satisfied with community safety.	3.445	0.734			0.854
	Q13	I’m quite satisfied with the overall evaluation.	3.499	0.720			0.880

We used the factor analysis method to extract the principal components as the residents’ community environmental perception. Three models of factor analysis, among which model 1 is residents’ perception of disorderly community environment, model 2 is residents’ perception of community attachment, and model 3 is residents’ satisfaction, were used. The *p* values of the Bartlett sphericity test probability of all three models are less than 0.001, indicating significant differences between the prior relational number matrix and the identity matrix.

The Kaiser-Meyer-Olkin (KMO) measure of sampling adequacy values are 0.803 (Model 1), 0.820 (Model 2), and 0.874 (Model 3). According to the KMO metric standard, the original variables are suitable for factor analysis. Interpretation of the total variance of the factor analysis shows the characteristic roots of the first factor are 2.643 (Model 1), 2.873 (Model 2), and 3.536 (Model 3). The characteristic root of the first factor in Model 1 explains 66.1% of the total variance of the four original variables. The characteristic root of the first factor in Model 2 explains 71.8% of the total variance of the four original variables. The characteristic root of the first factor in Model 3 explains 70.7% of the total variance of the five original variables. Finally, one principal factor was extracted from each of the three models.

#### 2.2.3 Analytic strategy

A binary logistic regression model was adopted to determine the association between independent variables and the community environmental perception. The empirical analysis model was constructed as follows:


(1) $$\ln(\frac{p_{i}}{1-p_{i}})=a+\sum_{k=1}^{k}\beta_{k}X_{\mathrm{ki}}$$

(2) or=p/(1−p)

In Equation 1, $p_{i}$ is the probability of intention to relocation of Respondent i in the future. $X_{\mathrm{ki}}$ is the factor that affects the probability of intention to relocation, i is the sample size, k is the total number of independent variables, *α* is the constant term independent of the independent variable, and $\beta_{k}$ is the partial regression coefficient. or is the odds ratio. The natural log of the odds ratio is ln(or), which can be expressed as a linear form of X and β . In Equations 1, 2, the odds ratio can be expressed in terms of exp. ( $\beta_{i}X_{i}$ ). The odds ratio measures the multiplier effect of the one-unit change of the explanatory variable $X_{i}$ to the ratio of contrasts, i.e., when the other variables are constant, an increase of one unit of Xi will cause the occurrence to expand/shrink by a factor of B.

Maximum likelihood method is used to estimate the parameters of the binary logistic regression by calculating the observed value of the chi-square of the model likelihood ratio and the probability *p*-value. The model is reasonable if the probability p-value is less than the given significance level, the null hypothesis should be rejected, and all regression coefficients in the current equation are zero at different times. The effect of the model was compared by −2 Log-likelihood, Pseudo R2 and Percentage Correct. The smaller the value of the former, the higher the degree of fit of the model. The larger the latter two values, the better the model-fitting effect. All analyses were performed using SPSS 22.0 software.

## 3 Results

### 3.1 Descriptive statistics

The variables are shown in Tables 1, 2. In terms of residents’ intention to relocation, among 1,568 respondents, 220 had the intention to relocation, accounting for 14.0%; 1,348 residents had no intention to relocation, accounting for 86.0%. In terms of the gender ratio of respondents, 53.3 percent were male, and 46.7 percent were female. In terms of marital status, married respondents accounted for 68.6%, while unmarried, divorced, or widowed respondents accounted for 31.4%. In terms of Hukou, local Hukou accounted for 43.6 percent, and non-local Hukou accounted for 56.4%. The respondents were 19 to 89 years old, and the average age was 38.030 years (SD = 14.016).

**Table 2:** Measurement and descriptive statistics of correlated variables.

Variable code	Variable name	Description	Minimum	Maximum	Mean	SD
*p*	Intention to relocation	Yes (1); No (0)	0.000	1.000	0.140	0.347
*X_1_*	Gender	Male (1); Female (2)	1.000	2.000	1.467	0.499
*X_2_*	Age	For adults only	19.000	89.000	38.030	14.016
*X_3_*	Marital status	Married (1) → Unmarried or divorced or widowed (2)	1.000	2.000	1.314	0.464
*X_4_*	Educational level	Uneducated (1) → Postgraduate or above (8)	1.000	8.000	4.262	1.608
*X_5_*	Household income	Below USD1458/month (1) → Over USD 11664/month (6)	1.000	6.000	1.750	1.079
*X_6_*	Hukou	Local (1) → non-local (2)	0.000	1.000	0.436	0.496
*X_7_*	Factor 1	Disorderly community environment	−2.363	2.604	0.000	1.000
*X_8_*	Factor 2	Community attachment	−3.492	2.211	0.000	1.000
*X_9_*	Factor 3	Satisfaction	−3.812	2.487	0.000	1.000

### 3.2 Model results

The analysis results of the binary logistic regression model are shown in Table 3. The *p*-value of the F-test statistic in the model is 0.000, indicating that the model is reasonable and that the independent and the corresponding dependent variables had a linear relationship. The variance inflation factors (VIF) of the independent variables are less than 5, indicating the absence of a serious collinearity problem among the variables.

**Table 3:** Binary logistic regression model of the residents’ intention to relocation.

Variable code	Variable name	B	SE	p	Exp (*B*)	Tol	VIF
*X*_0_	Constant	−0.707	0.633	0.264	0.493	--	--
*X*_1_	gender	−0.225	0.159	0.158	0.799	0.945	1.058
*X*_2_	Age	−0.026 **	0.009	0.003	0.975	0.563	1.776
*X*_3_	Marital status	−0.001	0.191	0.995	0.999	0.698	1.433
*X*_4_	Educational level	0.059	0.053	0.271	1.061	0.729	1.372
*X*_5_	Household income	0.034	0.070	0.631	1.034	0.874	1.144
*X*_6_	Hukou	−0.866***	0.185	0.000	0.421	0.804	1.243
*X*_7_	Factor 1	0.226**	0.077	0.004	1.254	0.980	1.020
*X*_8_	Factor 2	−0.238**	0.091	0.009	0.788	0.715	1.399
*X*_9_	Factor 3	−0.260**	0.087	0.003	0.771	0.739	1.354

χ^2^ = 119.040; −2 log likelihood = 1152.667; Nagelkerke R^2^ = 0.132; Cox & Snell R^2^ = 0.073; ***p < 0.001; **p < 0.01; *p < 0.05.

#### 3.2.1 Association between control variables and intention to relocation

The model includes six control variables, among which two variables (age and Hukou status) show significant associations with residents’ intention to relocate, while the other four (gender, marital status, educational level, and household income) do not. In the binary logistic regression model, a strong negative association between age and residents’ intention to relocate is observed (*B* = −0.026, *p* < 0.01, Exp *B* = 0.975), indicating that for each additional year of age, the odds of intending to relocate decrease by approximately 2.5%. This suggests that younger residents are more likely to consider relocation, possibly reflecting greater flexibility and mobility needs at earlier life stages. Similarly, Hukou status shows a significant negative association with relocation intention (*B* = −0.866, *p* < 0.001, Exp *B* = 0.421), meaning that residents with local Hukou have odds of intending to relocate that are 57.9% lower compared to those without local Hukou. This finding highlights the role of institutional factors, where possessing local registration may enhance residential stability due to better access to public services and housing benefits.

#### 3.2.2 Association between the perception of disorderly environment and intention to relocation

Residents’ perception of a disorderly community environment was synthesized through factor analysis into a comprehensive index (Factor 1). Logistic regression results reveal a significant positive association between Factor 1 and relocation intention (B = 0.226, *p* < 0.01, Exp(B) = 1.254). Specifically, with each one-unit increase in the perception of disorderly environment, the odds of intending to relocate increase by approximately 25.4%. This finding supports Hypothesis 1, suggesting that a perceived decline in the physical order and maintenance of the community acts as a strong “push” factor, prompting residents to consider moving to a more favorable environment.

#### 3.2.3 Association between community attachment and intention to relocation

Community attachment, synthesized from four survey items through factor analysis (Factor 2), shows a significant negative association with relocation intention (*B* = −0.238, *p* < 0.01, Exp *B* = 0.788). This means that for each one-unit increase in community attachment, the odds of intending to relocate decrease by approximately 21.2%. This finding supports Hypothesis 2 and emphasizes that emotional ties to the community, including neighborly relations and a sense of belonging, serve as important “retention” factors, discouraging residential mobility even when objective conditions may otherwise prompt relocation.

#### 3.2.4 Association between community satisfaction and intention to relocation

Residents’ satisfaction with their community, synthesized from five survey items through factor analysis (Factor 3), also exhibits a significant negative association with relocation intention (*B* = −0.260, *p* < 0.01, Exp *B* = 0.771). Each one-unit increase in community satisfaction reduces the odds of intending to relocate by approximately 22.9%. This result further supports Hypothesis 3, indicating that higher satisfaction with aspects such as housing conditions, infrastructure, and overall neighborhood environment substantially reduces residents’ willingness to relocate, underscoring the critical role of subjective evaluation in residential stability.

In order to systematically connect the theoretical assumptions to the empirical findings, a summary of hypothesis testing results is presented in Table 4. As shown, all three proposed hypotheses are supported by the logistic regression analysis. Specifically, higher perception of a disorderly environment significantly increases residents’ relocation intention, while stronger community attachment and higher satisfaction with the community environment are both associated with a decreased intention to relocate. These findings confirm the importance of subjective environmental perception in shaping residential mobility behaviors and provide solid empirical support for the conceptual framework proposed in this study.

**Table 4:** Summary of hypothesis testing results.

Hypothesis	Description	Result
H1	Higher perception of a disorderly community environment is positively associated with relocation intention.	Significant positive association (*B* = 0.226, *p* < 0.01)
H2	Stronger community attachment is negatively associated with relocation intention.	Significant negative association (*B* = −0.238, *p* < 0.01)
H3	Higher satisfaction with the community environment is negatively associated with relocation intention.	Significant negative association (*B* = −0.260, *p* < 0.01)

**Figure 2:**
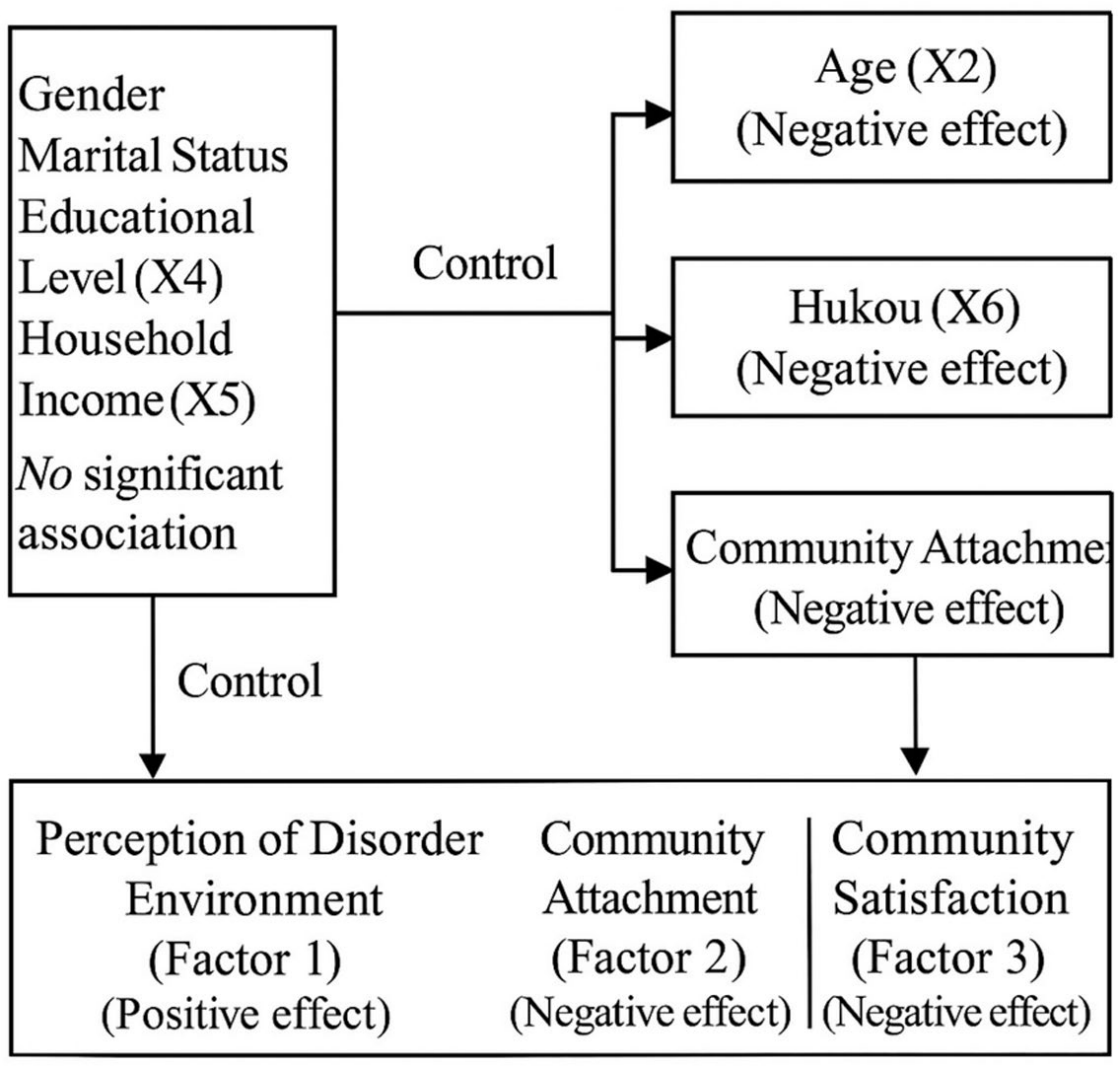
The conceptual framework.

Among the individual characteristics, gender, marital status, educational level, and household income were treated as control variables, as their associations with residential relocation intention were not statistically significant. In contrast, age and household registration (Hukou) exhibited significant negative effects, indicating that older individuals and those with local household registration were less likely to express relocation intentions. Additionally, three factors capturing residents’ perceptions of the community environment were identified as important influences: the perception of disorder in the environment (Factor 1) had a significant positive effect on relocation intention, while community attachment (Factor 2) and community satisfaction (Factor 3) both showed significant negative effects. Overall, the framework highlights how personal demographics and subjective evaluations of the community jointly shape residents’ propensity to consider relocation (Figure 2).

#### 3.2.5 Robustness check

A random sampling method was employed to test the robustness of the binary logistic regression model, and the results indicate that the model demonstrates good robustness. The robustness check involved five models, each based on a sampling proportion of 80.0% (1,254 samples). Regarding the age variable, the regression coefficients across the five models generally ranged between −0.032 and-0.021, all reaching a moderate level of significance. For the household registration variable, the coefficients ranged between −0.931 and −0.835, all exhibiting a high level of significance. As for the three variables reflecting residents’ perceptions of the community environment, except for Factor 1 in Model 1, which was marginally insignificant (*p*-value = 0.081), the other three variables consistently showed statistical significance across all models, with relatively small fluctuations in the range of their regression coefficients (see Table 5).

**Table 5:** Robustness checks of binary logistic regression model.

Variable code	Variable name	Model 1	Model 2	Model 3	Model 4	Model 5
*X*_0_	Constant	−0.646	−0.398	−0.733	−0.398	−0.174
*X*_1_	Gender	−0.216	−0.289	−0.221	−0.168	−0.162
*X*_2_	Age	−0.026**	−0.028**	−0.021*	−0.032**	−0.030**
*X*_3_	Marital status	−0.158	−0.228	0.043	−0.025	−0.101
*X*_4_	Educational level	0.106	0.040	0.060	0.015	0.006
*X*_5_	Household income	0.031	0.109	−0.066	0.031	0.004
*X*_6_	Hukou	−0.916***	−0.878***	−0.931***	−0.880***	−0.835***
*X*_7_	Factor 1	0.149	0.235**	0.284***	0.275**	0.266 **
*X*_8_	Factor 2	−0.249**	−0.340***	−0.259*	−0.305**	−0.227*
*X*_9_	Factor 3	−0.316**	−0.212*	−0.225*	−0.255*	−0.267**

***means significants in 99%, **means significants in 95%, and *means significants in 90%.

## 4 Discussion

This study aimed to contribute to the analytical framework of residents’ intention to relocation in the non-Western context from the perspective of community environmental perception. Overall, a significant association was observed between residents’ relocation intention and their subjective perceptions of the community environment. Specifically, residents who perceived a more disorderly environment showed a higher intention to relocate, while those with stronger emotional attachment and higher satisfaction toward their communities were less inclined to move. These findings are consistent with our initial hypotheses and collectively highlight the pivotal role of subjective community environmental perception in shaping relocation behaviors.

It is important to acknowledge, however, that the data used in this study were collected in 2016. Given the rapid pace of urban development, societal transformation, and policy changes over the past decade, residents’ community environmental perceptions and relocation intentions may have evolved over time. This temporal gap may affect the direct applicability of the findings to the current urban context. Nevertheless, the fundamental relationships identified between environmental disorder perception, community attachment, satisfaction, and relocation intentions remain theoretically robust and provide meaningful insights into the micro-mechanisms of residential mobility. Future research could benefit from incorporating updated datasets or adopting longitudinal approaches to capture the dynamic changes in urban living environments and their influence on residents’ relocation decisions.

Existing studies on residential relocation often adopt the residential relocation that has already happened, ignoring the emotional needs of those who intend to move but are not allowed by objective conditions. The explanation of the causes of residential relocation focuses more on the matching relationship between the objective housing needs of the respondents and the objective environment of the community and less on the influencing factors at the supervisor level, such as the perception of disorderly environment, place attachment, and community satisfaction (36–38). To better explain the association between community environmental perception and intention to relocation, this study conducted a statistical analysis of why residents moved to the community, why they stayed in the community, and why they wanted to move out of the community (39).

### 4.1 Why did the respondents choose their community?

The main reasons for the respondents to choose their current community are rigid needs and passive choices, while community environmental perception and emotional needs had a lesser role. Among them, the reason of individuals and other family members work convenience accounts for the highest proportion (QC4). Economic reasons, such as cheap housing and rent, ranked second (QC1). Convenient transportation ranked third (QC6). The first three reasons accounted for 62.6%, and other reasons were mainly life convenience or life node reasons (such as marriage and childbirth), while environmental perception-related reasons accounted for a relatively low proportion (see Table 6).

**Table 6:** Reasons for residents to choose their current community.

Question code	Description	Number	Percentage (%)
QC1	The house price or rent is cheap.	348	22.2
QC2	House demolition and resettlement	29	1.8
QC3	Housing allocated by the government as welfare	89	5.7
QC4	It is convenient for me and my family to work.	400	25.5
QC5	The supporting facilities are perfect and convenient.	155	9.9
QC6	The community has convenient transportation.	234	14.9
QC7	The community is very safe.	105	6.7
QC8	The house used to be rented but is now bought.	30	1.9
QC9	Marriage	57	3.6
QC10	Childbirth	6	0.4
QC11	Children go to school	34	2.2
QC12	Other reasons	81	5.2

Total samples number = 1,568.

### 4.2 Why do respondents plan to stay?

In the sample, residents lived in their communities for a minimum of 3 months, a maximum of 79 years, an average of 10.508 years, and a standard deviation of 12.122 years. A total of 1,348 respondents plan to continue living in their existing communities. The reasons for staying in the current community can be divided into four categories. The first is due to the convenience of work and life (QS3, QS3 and QS3), accounting for 48.1%. The second is that they are satisfied with the neighborhood relationship and attachment to the community, accounting for 30.7%. The third reason is that while they are satisfied with the social environment, they cannot afford to buy a house, accounting for 18.7%; The fourth category was other reasons (for example, other family members do not want to move), accounting for 2.4% (Table 7).

**Table 7:** Reasons why the respondent did not plan for relocation.

Question code	Description	Number	Percentage (%)
QS1	Not satisfied with the community but cannot afford to buy a house	252	18.7
QS2	I’m satisfied with the neighborhood and attached to the community.	414	30.7
QS3	The community is convenient for me and my family to work.	460	34.1
QS4	The community is convenient for children to go to school.	111	8.2
QS5	Community public service facilities make life convenient.	78	5.8
QS6	Other reasons	33	2.4

Total samples number = 1,348.

### 4.3 Why do residents plan to leave?

A total of 220 respondents planned to leave their communities. There are 11 options for the main reasons, which can be summarized into 6 aspects. The highest reason was the convenience of existing communities, such as QL2, QL4 and QL9, accounting for 40.9%. The second highest reason is the respondents’ satisfaction with the community environment, accounting for 24.5%. The options for economic reasons included QL6 and QL10, which accounted for 11.8%. The reasons for emotional belonging included QL6 and QL5, which accounted for 14.1%. The reasons for the life node category include marriage and childbirth, which accounted for 2.7%. Other reasons (QL11) accounted for 5.9% (Table 8).

**Table 8:** Reasons why the respondents plan to relocation.

Question code	Description	Number	Percentage (%)
QL1	I am not satisfied with the community environment and want to improve my living environment.	54	24.5
QL2	Make it convenient to work for myself and my family	53	24.1
QL3	Community away from relatives and friends	13	5.9
QL4	Community life is not convenient	18	8.2
QL5	I now live as a renter and want to own my house	18	8.2
QL6	The rent in my current residence is too high.	14	6.4
QL7	I’m getting married.	5	2.3
QL8	I’m ready to have a baby.	1	0.5
QL9	For the convenience of their children’s schooling	19	8.6
QL10	I now live in a house for investment to make money	12	5.5
QL11	Other reasons	13	5.9

Total samples number = 220.

## 5 Conclusion

This study aims to analyze the association between community environmental perception and residents’ intention to relocation from the perspective of environmental perception. The main findings are as follows. (1) Many important life node events (such as marriage and birth of children) of residents are related to age. Thus, a significant association between the intention to move and age can be observed. (2) Under China’s special social and cultural background, a association exists between having a local Hukou and residents’ residential relocation intention. (3) Residents’ community environmental perception can be subdivided into three dimensions: disorderly environment, community attachment and satisfaction, each of which significantly correlates with residents’ intention to live and move. (4) From the perspective of association significance, satisfaction and residential relocation intention had the highest degree of association, followed by disorderly environment, while community attachment and residential relocation intention had the lowest association.

Furthermore, the findings highlight the critical importance of subjective community environmental perceptions in shaping relocation behavior, especially in rapidly urbanizing and institutionally unique contexts like China. Traditional urban renewal and community development policies have primarily focused on improving objective environmental conditions. However, this study suggests that policymakers should also pay greater attention to enhancing residents’ emotional attachment and subjective satisfaction with their communities, which may be equally or even more effective in reducing residential instability and fostering long-term community cohesion.

The independent variable selected is residents’ community environmental perception rather than the objective community environment. Residents’ intention to relocation is an important concern in urban geography. The existing literature often explains the residential relocation intention from the perspective of objective community environment (40, 41). For example, residents move from communities with poor living conditions to communities with better living conditions to improve their living environment (42). Undeniably, objective community environment is the basis of subjective community environment. However, due to differences in individual social and demographic attributes, subjective community environment is not equivalent to objective community environment, and residents in the same community have different community environmental perceptions. Therefore, the study of residents’ intention to relocation from the perspective of community environmental perception is a useful supplement to the existing literature.

In the screening process of control variables, this study considers the influence of demographic characteristics on residents’ intention to relocation and considers variable elements in non-western cultural backgrounds, such as Hukou. In terms of demographic characteristics indicators, the existing literature believes that residential relocation matches the ability and demand of individuals at different life stages (43). For example, young people who have just started working usually transition to rental houses with poorer conditions, and the frequency of residential relocation is relatively high. After family stability (such as marriage and children’s birth), they will buy a house, and their willingness to move will be significantly reduced. When they retire, they move from their own house to a rental house after retirement. The conclusion of this study shows that the relationship between age and intention to relocation is significant and is consistent with the conclusion of previous studies. In addition, Hukou, a variable index with unique Chinese characteristics, is also included in the control variables. The results show that the association level between household registration and residential relocation intention is the highest among the selected variables. This conclusion is a good addition to the explanation of residential relocation intention.

Residents’ intention to relocation is highly correlated with their community environmental perception. With the further development of the economy and society and the further improvement of China’s housing security system, the degree of restriction of economy and housing supply on residential relocation will be further reduced, and the subjective intention to relocation will be changed into the objective behavior of residential relocation to a greater extent. Therefore, reducing the degree of disorder in the community environment and improving residents’ satisfaction and emotional attachment to the community environment is of certain value for constructing the community environment.

In the future, further research should be conducted based on this study to explore the differences in community environmental perceptions and migration willingness among subgroups. Studies have shown that African immigrants, as one of the most representative immigrant subgroups, have significant differences in their perceptions of personal safety and the environment compared to local residents (44). Although this study focused on the direct associations between individual factors and relocation intention, future research could explore potential interaction effects among these factors. For example, the influence of perceived environmental disorder on relocation intention might be moderated by the level of community attachment or satisfaction. Residents with strong emotional ties to their communities may be less sensitive to environmental disorder when forming relocation intentions, compared to those with weaker ties. Investigating such interaction effects could provide deeper insights into the complex decision-making processes behind residential mobility.

Therefore, exploring the psychological and environmental perception differences in housing relocation decisions among different subgroups helps to better understand the unique needs and responses of these groups when faced with housing policies and social migration. These differences can influence their acceptance, adaptability, and willingness to relocate to new living environments. By identifying and analyzing the distinct needs of these subgroups in terms of safety perception, community belonging, and quality of life, policymakers can design more inclusive policies that ensure all groups, especially immigrant and socially vulnerable groups, can equally benefit from housing policies. Additionally, this can help reduce social exclusion, promote integration and harmony among different cultural groups, and ensure that housing policies effectively support the overall stability and sustainable development of society. Moreover, the analysis in this study is primarily based on cross-sectional data rather than time-series data. Therefore, future research could further enhance the data chain of the paper.

## Data Availability

The original contributions presented in the study are included in the article/supplementary material, further inquiries can be directed to the corresponding author.

## Footnotes

^1^ https://lwzb.gzstats.gov.cn/

## References

[1] Michielin F Mulder CH. Family events and the residential mobility of couples. Environ Plann A: Econ Space. (2008) 40:2770–90. doi: 10.1068/a39374

[2] Hooimeijer P Knaap B. From flows of people to networks of behaviour. Ned Geogr Stud. (1994) 173:178–87.

[3] Lee ES. A theory of migration. Demography. (1966) 3:47–57. doi: 10.2307/2060063

[4] Speare A. Residential satisfaction as an intervening variable in residential mobility. Demography. (1974) 11:173–88. doi: 10.2307/2060556, PMID: 21274806

[5] Morris T Manley D Sabel CE. Residential mobility: towards progress in mobility health research. Prog Hum Geog. (2018) 42:112–33. doi: 10.1177/0309132516649454, PMID: 30369706 PMC6187834

[6] Jie G Suhong Z Xiaopei Y. The space-time paths of residential mobility in Guangzhou from a perspective of life course. Geogr Res. (2013) 32:157–65. doi: 10.11821/yj2013010016

[7] Clark WAV Lisowski W. Decisions to move and decisions to stay: life course events and mobility outcomes. Housing Stud. (2017) 32:547–65. doi: 10.1080/02673037.2016.1210100

[8] Böheim R Taylor MP. Tied down or room to move? Investigating the relationships between housing tenure, employment status and residential mobility in britain. Scot J Polit Econ. (2002) 49:369–92. doi: 10.1111/1467-9485.00237

[9] Brimblecombe N Dorling D Shaw M. Migration and geographical inequalities in health in britain. Soc Sci Med. (2000) 50:861–78. doi: 10.1016/S0277-9536(99)00371-8 10695983

[10] Grundy EMD Fox AJ. Migration during early married life. Eur J Popul. (1985) 1:237–63. doi: 10.1007/BF01796934, PMID: 12340531

[11] Kulu H. Migration and fertility: competing hypotheses re-examined. Eur J Popul/Rev eur Démogr. (2005) 21:51–87. doi: 10.1007/s10680-005-3581-8

[12] Mulder CH Wagner M. Migration and marriage in the life course: a method for studying synchronized events. Eur J Popul. (1993) 9:55–76. doi: 10.1007/BF01267901, PMID: 12344903

[13] Clark WAV Huang Y. The life course and residential mobility in british housing markets. Environ Plann A. (2003) 35:323–39. doi: 10.1068/a3542

[14] Feijten P Van Ham M. The impact of splitting up and divorce on housing careers in the UK. Housing Stud. (2010) 25:483–507. doi: 10.1080/02673031003711477

[15] De Jong GF. Expectations, gender, and norms in migration decision-making. Popul Stud. (2000) 54:307–19. doi: 10.1080/713779089, PMID: 28489514

[16] Van Ham M Feijten P. Who wants to leave the neighbourhood? The effect of being different from the neighbourhood population on wishes to move. Environ Plann A. (2008) 40:1151–70. doi: 10.1068/a39179, PMID: 32306168

[17] Bolt G Van Kempen R Van Weesep J. After urban restructuring: relocations and segregation in dutch cities. Tijdschr Econ Soc Ge. (2009) 100:502–18. doi: 10.1111/j.1467-9663.2009.00555.x

[18] Clark WAV Van Ham M Coulter R. Spatial mobility and social outcomes. J Housing Built Environ. (2014) 29:699–727. doi: 10.1007/s10901-013-9375-0

[19] He J Li C Huang J Liu D Yu Y. Modeling urban spatial expansion considering population migration interaction in Ezhou, Central China. J Urban Plann Dev. (2019) 145:5019003. doi: 10.1061/(ASCE)UP.1943-5444.0000503

[20] Katsarski N. Factors determining migration of the population. Knowl Int J. (2019) 30:1729–33. doi: 10.35120/kij30061729K

[21] Wei W Ren X Guo S. Evaluation of public service facilities in 19 large cities in China from the perspective of supply and demand. Land. (2022) 11:149. doi: 10.3390/land11020149

[22] Tsang F Rohr C. The impact of migration on transport and congestion. Santa Monica, CA: RAND Corporation (2011). Available online at: https://www.jstor.org/stable/10.7249/j.ctt3fh24x (Accessed January 5, 2025)

[23] Cochrane W Poot J. Effects of immigration on local housing markets In: Kourtit K Newbold B Nijkamp P Partridge M, editors. The economic geography of cross-border migration. Footprints of regional science. Cham: Springer International Publishing (2021). 269–92.

[24] Chen A. China’s urban housing market development: problems and prospects. J Contemp China. (1998) 7:43–60. doi: 10.1080/10670569808724304

[25] Foell A Fowler PJ Purnell JQ Nebbitt V Jabbari J Chun Y. Choice and opportunity: housing relocation, neighborhood change, and family well-being in the south city choice neighborhoods initiative (CNI) in Memphis. TN Housing Policy Debate. (2024):1–27. doi: 10.1080/10511482.2023.2291347, PMID: 40330611

[26] Wells NM Harris JD. Housing quality, psychological distress, and the mediating role of social withdrawal: a longitudinal study of low-income women. J Environ Psychol. (2007) 27:69–78. doi: 10.1016/j.jenvp.2006.11.002

[27] Fussell E Lowe SR. The impact of housing displacement on the mental health of low-income parents after hurricane Katrina. Soc Sci Med. (2014) 113:137–44. doi: 10.1016/j.socscimed.2014.05.025, PMID: 24866205 PMC4096953

[28] Shi P Vanclay F Yu J. Post-resettlement support policies, psychological factors, and farmers’ homestead exit intention and behavior. Land. (2022) 11:237. doi: 10.3390/land11020237

[29] Tao Y. Linking residential mobility with daily mobility: a three-wave cross-lagged panel analysis of travel mode choices and preferences pre–post residential relocation in the Netherlands. Urban Stud. (2024) 61:273–93. doi: 10.1177/00420980231181049

[30] Saucy A Ortega N Tonne C. Residential relocation to assess impact of changes in the living environment on cardio-respiratory health: a narrative literature review with considerations for exposome research. Environ Res. (2024) 244:117890. doi: 10.1016/j.envres.2023.117890, PMID: 38081343

[31] Mengyong W. Research on community identity, emotional structure of the environment, and urban morphogenetic mechanism: based on the measurement and evaluation of caoyang xincun in shanghai. City Plan Rev. (2018) 42:43–54.

[32] Kienast-von Einem C Panter J Ogilvie D Reid A. Exploring residential relocation– differences between newcomers and settled residents in health, travel behaviour and neighbourhood perceptions. Health Place. (2024) 87:103254. doi: 10.1016/j.healthplace.2024.103254, PMID: 38701677

[33] Brazil N Clark WAV. Residential mobility and neighborhood inequality during the transition to adulthood. Urban Geogr. (2019) 40:938–63. doi: 10.1080/02723638.2018.1506614

[34] Jing F Liu L Zhou S Song G. Examining the relationship between Hukou status, perceived neighborhood conditions, and fear of crime in Guangzhou, China. Sustain For. (2020) 12:9614. doi: 10.3390/su12229614

[35] Colas M Ge S. Transformations in China’s internal labor migration and Hukou system. J Labor Res. (2019) 40:296–331. doi: 10.1007/s12122-019-9283-5

[36] Scannell L Gifford R. Defining place attachment: a tripartite organizing framework. J Environ Psychol. (2010) 30:1–10. doi: 10.1016/j.jenvp.2009.09.006

[37] Scannell L Gifford R. The experienced psychological benefits of place attachment. J Environ Psychol. (2017) 51:256–69. doi: 10.1016/j.jenvp.2017.04.001

[38] Lewicka M. Place attachment, place identity, and place memory: restoring the forgotten city past. J Environ Psychol. (2008) 28:209–31. doi: 10.1016/j.jenvp.2008.02.001

[39] Li C He S. Too privileged to move? Neighbourhood perception and relocation intention in China’s gated communities. Tijdschr Econ Soc Ge. (2024) 115:691–705. doi: 10.1111/tesg.12616

[40] Coulter R Van Ham M. Following people through time: an analysis of individual residential mobility biographies. Housing Stud. (2013) 28:1037–55. doi: 10.1080/02673037.2013.783903

[41] Kim JH. Residential and job mobility: interregional variation and their interplay in US metropolitan areas. Urban Stud. (2014) 51:2863–79. doi: 10.1177/0042098013514496

[42] Wang F Wang D. Changes in residential satisfaction after home relocation: a longitudinal study in Beijing, China. Urban Stud. (2020) 57:583–601. doi: 10.1177/0042098019866378

[43] Mulder CH. The housing consequences of living arrangement choices in young adulthood. Housing Stud. (2003) 18:703–19. doi: 10.1080/02673030304255

[44] Song G Liu L He S Cai L Xu C. Safety perceptions among African migrants in Guangzhou and Foshan. China Cities. (2020) 99:102624. doi: 10.1016/j.cities.2020.102624, PMID: 40348662

